# Comparisons among the Ultrasonography Prediction Model, Real-Time and Shear Wave Elastography in the Evaluation of Major Salivary Gland Tumors

**DOI:** 10.3390/diagnostics12102488

**Published:** 2022-10-14

**Authors:** Ping-Chia Cheng, Wu-Chia Lo, Chih-Ming Chang, Ming-Hsun Wen, Po-Wen Cheng, Li-Jen Liao

**Affiliations:** 1 Department of Otolaryngology Head and Neck Surgery, Far Eastern Memorial Hospital, No. 21, Sec. 2, Nanya S. Rd., Banqiao Dist., New Taipei City 220, Taiwan; 2 Head and Neck Cancer Surveillance and Research Study Group, Far Eastern Memorial Hospital, New Taipei City 220, Taiwan; 3 Department of Biomedical Engineering, National Yang Ming Chiao Tung University, Taipei 112, Taiwan; 4 Department of Communication Engineering, Asia Eastern University of Science and Technology, New Taipei City 220, Taiwan; 5 Graduate Institute of Medicine, Yuan Ze University, Taoyuan 320, Taiwan; 6 Department of Electrical Engineering, Yuan Ze University, Taoyuan 320, Taiwan; 7 Medical Engineering Office, Far Eastern Memorial Hospital, New Taipei City 220, Taiwan

**Keywords:** major salivary gland tumor, ultrasonography, shear wave elastography, real-time elastography, ultrasound-guided fine needle aspiration

## Abstract

We aimed to validate the diagnostic accuracy of a novel sonographic scoring model and compare it with other methods in the evaluation of malignant major salivary gland tumors. We enrolled 138 patients who received neck ultrasound (US) with fine needle aspiration (FNA) and further operations or core needle biopsies for major salivary gland tumors from June 2015 to October 2021. The sonographic scoring model was presented as 2.08 × (vague boundary) + 1.75 × (regional lymphadenopathy) + 1.18 × (irregular or speculated shape) + 1.45 × (absence of posterior acoustic enhancement) + 2.4 × (calcification). We compared the diagnostic accuracy of the sonographic scoring model with shear wave elastography (SWE), real-time elastography (RTE), and US-FNA cytology for differentiating between benign and malignant lesions. The sensitivity, specificity, and accuracy were 58%, 89%, and 85% for the sonographic scoring model, 74%, 62%, and 64% for the SD of SWE with optimal cutoff value of 31.5 kPa, 69%, 70%, and 70% for the 4-point scoring system of RTE, and 74%, 93%, and 91% for US-FNA cytology, respectively. The sonographic scoring model is feasible as assistance in the evaluation of major salivary gland tumors. US-FNA cytology remains the tool of choice in diagnosing malignant salivary gland tumors.

## 1. Introduction

Salivary glands secrete saliva, which aids in digestion, keeps the mouth moist, and supports healthy teeth [1]. Salivary gland tumors are rare and can be benign or malignant. Sometimes, it is difficult to differentiate the pathology before surgical intervention.

Ultrasound (US) is widely used in the evaluation of neck tumors, including major salivary gland tumors. Usually, otolaryngologists and head and neck surgeons use grayscale and power Doppler US to check the characteristics of salivary gland tumors. There are several sonographic features of malignant salivary gland tumors [2,3,4]. However, no single parameter is adequate for diagnosing malignancy. In our previous study [2], we developed a sonographic scoring model based on the US characteristics as 2.08 × (vague boundary) + 1.75 × (regional lymphadenopathy) + 1.18 × (irregular or speculated shape) + 1.45 × (absence of posterior acoustic enhancement) + 2.4 × (calcification). The variable was counted as 1 if the above characteristic was positive and counted as 0 if negative. The cutoff value for classifying malignancy was greater than or equal to 3. For diagnosing malignant salivary gland tumors, it showed high specificity (94%) and accuracy (90%).

US elastography (USE) is a novel imaging tool for the evaluation of tissue elasticity and was first described in 1990 [5]. USE can be classified into strain elastography (SE) and shear wave elastography (SWE) by measurement of the tissue’s different physical quantities. SE is obtained by parallel displacement of the target after cycling compression and relaxation by the US transducer; SWE is obtained by the perpendicular shear wave, which is generated by the higher intensity pulse of the US transducer [6]. Many studies have applied SE in the evaluation of salivary gland tumors. One meta-analysis included nine studies of real-time elastography (RTE), the major type of SE [7]. The results showed that for differentiating between benign and malignant lesions, the pooled sensitivity and specificity were 76% (95% CI, 65% to 85%) and 73% (95% CI, 62% to 81%), respectively. The area under the receiver operating characteristic (ROC) curve was 0.81. However, they concluded that compared with RTE in the evaluation of breast and liver lesions, the accuracy of RTE in diagnosing malignant salivary gland lesions was low [7]. On the other hand, few studies have used SWE to evaluate salivary gland tumors, and the results are equivocal [8,9,10,11].

In this study, we aimed to (1) validate the diagnostic accuracy of the sonographic scoring model for major salivary gland tumors with another case series, (2) report our experience in using SWE to evaluate the major salivary gland tumors, and (3) compare the diagnostic accuracy of the sonographic scoring model, SWE, RTE, and US-guided fine needle aspiration (US-FNA) cytology in the evaluation of malignant major salivary gland tumors.

## 2. Materials and Methods

### 2.1. Ethical Considerations

This retrospective study was performed in accordance with the Declaration of Helsinki and with approval of the institutional ethical review board (IRB No. 110153-E and No.111199-E). The study did not influence the patients’ treatment or outcome.

### 2.2. Inclusion Criteria

We collected patients diagnosed from June 2015 to October 2021 at a tertiary medical center. In our hospital, patients with suspicious major salivary gland tumors were referred for neck US, which was performed by two experienced otolaryngologists (Li-Jen Liao and Wu-Chia Lo). US-FNA was performed in all major salivary gland tumors with one single aspiration under the free-hand technique at the US room. Core needle biopsy (CNB) was performed with 18-gauge cutting biopsy needle (Temno biopsy system, Allegiance Healthcare Corporation, McGaw Park, IL, USA) and usually one single biopsy when lymphoma was suspected or failure of diagnosis by FNA. Adult patients (aged 20 years or older) who had pathological reports and who either underwent further operations or core needle biopsies were included. The pathological reports, which served as the gold standard, were classified into malignancy and benignity. Benign salivary gland tumors were further classified into pleomorphic adenoma (PA), Warthin’s tumor (WT), and other benign tumors (BTs). Those who did not receive SWE were excluded.

### 2.3. Outcome Assessment

**Figure 1:**
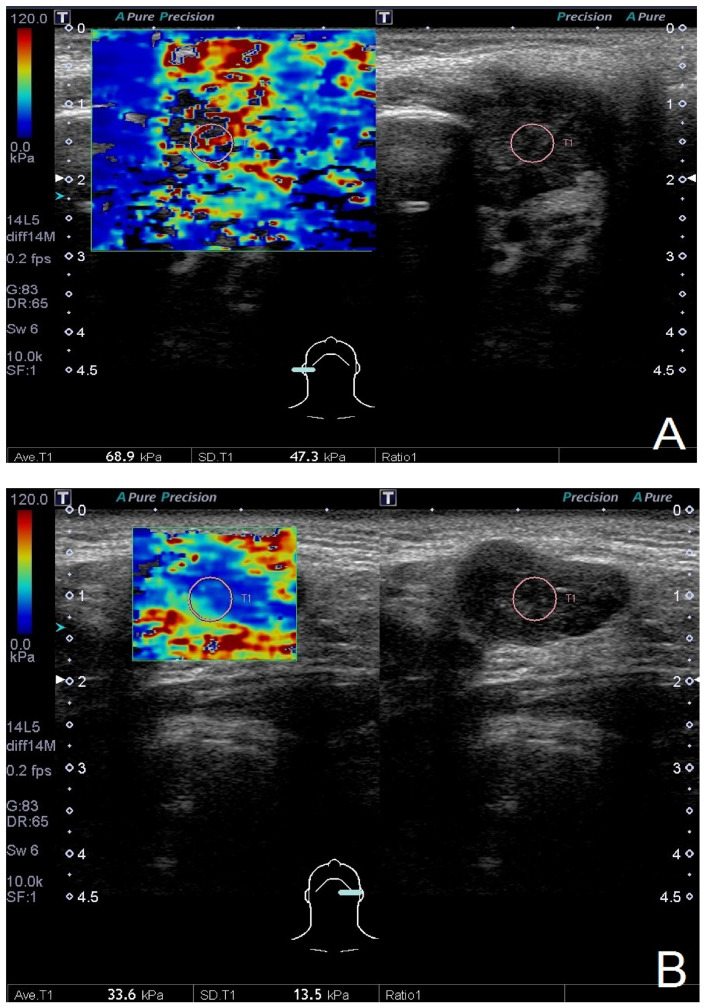
The measurement of shear wave elastography with a 5-mm circular region of interest. *Note.* (**A**) A 21-year-old female had a right parotid tumor for several months. The average and standard deviation of elasticity were 68.9 kPa and 47.3 kPa, respectively. The pathological report showed mucoepidermoid carcinoma. (**B**) A 59-year-old male had a left parotid tumor for several months. The average and standard deviation of elasticity were 33.6 kPa and 13.5 kPa, respectively. The pathological report showed Warthin’s tumor.

B-mode US and USE were performed without contrast using a 5–14 MHz linear-array transducer, the Toshiba Aplio 500 (Canon Medical Systems, Tochigi-ken, Japan). The sonographic scoring model was calculated based on the characteristics under B-mode US. A score greater than or equal to 3 was classified as malignant. The shear wave was generated after the acoustic pulse and recorded as the shear wave velocity (m/s), which was further converted to Young’s modulus (kPa) [12,13]. We placed a 5-mm circular region of interest (ROI) on the stiffest area of the salivary gland tumor by visual inspection. The Young’s modulus of the 5-mm ROI was calculated automatically and displayed as the average and standard deviation (Figure 1). We recorded SWE as the Young’s modulus (kPa). The average and standard deviation (which represents the heterogeneity) of elasticity with the optimal cutoff value were compared according to pathological reports.

We also compared the diagnostic accuracy of the sonographic scoring model, SWE, 4-point scoring system of RTE, and US-FNA cytology in the evaluation of malignant major salivary gland tumors. During the RTE examination, images were superimposed and adjacent to the grayscale US as a dual-panel image. The sonographers followed light pressure compression with repeated decompression until nearly identical sizes and color distributions of the region of interest in several consecutive images were obtained. The strain quality indicator was determined by manually appropriate compression adjustments to obtain smooth sine waves and avoid under- or overcompression [14]. The RTE images are presented with colors; blue represents the stiffest area, and green represents the softest area. We used the 4-point scoring system to classify RTE, and a score of 3 or 4 was classified as malignancy. A score of 1 represents almost soft (almost green within the lesion), a score of 2 represents mostly soft (green more than blue), a score of 3 represents mostly stiff (blue more than green), and a score of 4 represents almost stiff (almost blue). Using FNA cytology, the cytological report of atypia [15,16,17,18,19], suspicious malignancy or malignancy was classified as suspicious malignancy.

### 2.4. Statistical Analysis

Statistical analysis was performed using STATA software, version 12.0 (Stata Corporation, College Station, TX, USA). The clinical characteristics and pathological reports are displayed as the mean and standard deviation (SD) or number and percent (%). The detailed list of elasticity is displayed as the median and interquartile range (IQR). Comparisons of SWE were conducted using the Wilcoxon rank-sum test and Kruskal-Wallis test. We also calculated the area under the ROC curve (AUC) of the standard deviation of SWE to determine its diagnostic accuracy in the diagnosis of malignant salivary gland tumors.

## 3. Results

A total of 138 patients were included in this study who were predominantly male (60% [83/138]). The characteristics are summarized in Table 1. Pathological reports revealed 119 BTs (49 WTs, 48 Pas, and 22 other BTs) and 19 malignant tumors. The mean (SD) age was 53 (14) years, ranging from 20 to 94 years. The mean (SD) long axis and short axis of the tumors were 2.5 (1.0) cm and 1.7 (0.7) cm, respectively.

We first validated the sonographic scoring model in the diagnosis of malignant salivary gland tumors, and the sensitivity, specificity, and accuracy were 58% (34% to 80%), 89% (84% to 95%), and 85% (79% to 91%), respectively. We also arranged the ROC analysis of the sonographic scoring model, and the AUC was 0.82.

**Figure 2:**
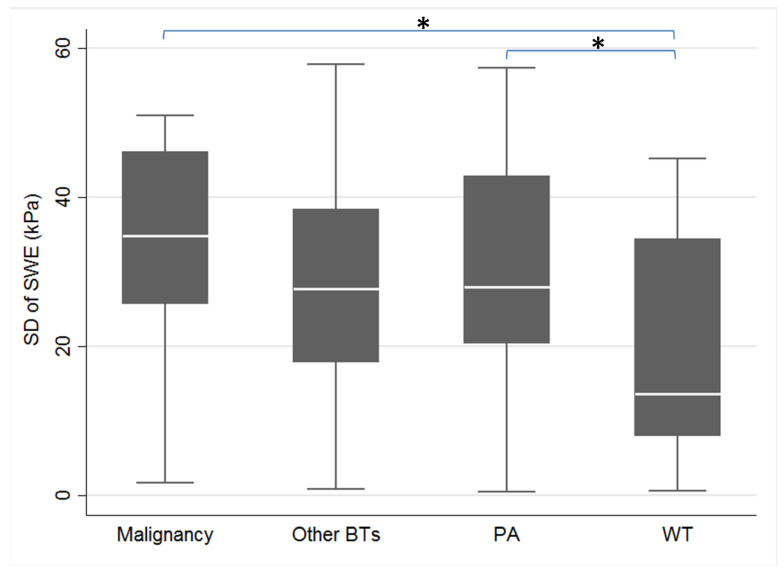
Comparison of the standard deviation of elasticity among different pathologies with Young’s modulus. Abbreviations: BT, benign tumor; PA, pleomorphic adenoma; SD, standard deviation; SWE, shear wave elastography; WT, Warthin’s tumor. *Note.* The standard deviation of elasticity of malignant tumors, pleomorphic adenoma, other benign tumors, and Warthin’s tumor showed significant differences under the Kruskal-Wallis test (median ± IQR, 34.8 ± 20.4, 28 ± 22.4, 27.7 ± 20.5, and 13.7 ± 26.4, *p* < 0.01). Comparisons within the group showed a significant difference between malignant tumor and Warthin’s tumor (*p* < 0.01) and between pleomorphic adenoma and Warthin’s tumor (*p* < 0.01), but no difference between malignant tumor and pleomorphic adenoma (*p* = 0.21), between malignant tumor and other benign tumor (*p* = 0.15), between pleomorphic adenoma and other benign tumor (*p* = 0.62) and between Warthin’s tumor and other benign tumor (*p* = 0.06). * Statistical significance, *p* < 0.05.

**Figure 3:**
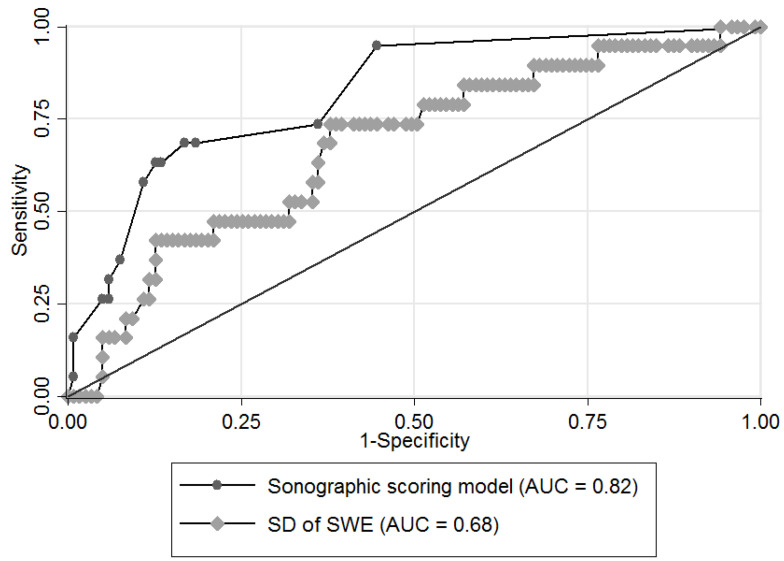
Comparison of ROC curves of the sonographic scoring model and the standard deviation of elasticity for differentiating malignancy from benignancy. Abbreviation: ROC, receiver operating characteristic; SD, standard deviation; SWE, shear wave elastography; AUC, area under the ROC curve. *Note.* For diagnosing malignant salivary gland tumors, the AUCs were 0.82 in the sonographic scoring model and 0.68 in the SD of elasticity.

Second, we analyzed the diagnostic accuracy of SWE. The elasticity (Young’s modulus) of SWE is shown in Table 2. There was no significant difference in average elasticity between benign and malignant tumors (median ± IQR, 49.4 ± 40.3 vs. 61.8 ± 32.7 kPa, *p* = 0.31), but there was a significant difference in standard deviation of elasticity between benign and malignant tumors (median ± IQR, 25.9 ± 25 vs. 34.8 ± 20.4 kPa, *p* = 0.01). The standard deviation of elasticity among malignant tumors, PAs, other BTs and WTs also showed a significant difference under the Kruskal-Wallis test (median ± IQR, 34.8 ± 20.4, 28 ± 22.4, 27.7 ± 20.5, and 13.7 ± 26.4 kPa, *p* < 0.01) (Figure 2). However, for differentiating malignancy from benignancy, the AUC of the standard deviation of elasticity was only 0.68 (Figure 3). The optimal cutoff value was 31.5 kPa, and the sensitivity, specificity, and accuracy were 74% (95% CI: 54% to 94%), 62% (54% to 71%), and 64% (56% to 72%), respectively (Table 3).

We further compared the accuracy of the sonographic scoring model, SWE, 4-point scoring system of RTE, and US-FNA cytology in the diagnosis of malignant salivary gland tumors, and the results are summarized in Table 3. Using the 4-point scoring system of RTE, the sensitivity, specificity, and accuracy were 69% (46% to 92%), 70% (61% to 79%), and 70% (62% to 78%), respectively. Using US-FNA cytology, the sensitivity, specificity, and accuracy were 74% (54% to 94%), 93% (89% to 98%), and 91% (86% to 96%), respectively. Among these four methods, US-FNA cytology reported the highest specificity (93% [89% to 98%]) and accuracy (91% [86% to 96%]).

## 4. Discussion

This is a comprehensive study comparing the sonographic scoring model, elastography, and US-FNA cytology in assessing major salivary gland tumors. In this study, we validated the sonographic scoring model in the diagnosis of malignant salivary gland tumors, and the results showed good specificity [89% (84% to 95%)] and accuracy [85% (79% to 91%)]. In addition, this was also the largest case series using SWE in the evaluation of major salivary gland tumors. There was a significant difference in the standard deviation (SD) of elasticity between benign and malignant tumors (median ± IQR, 25.9 ± 25 vs. 34.8 ± 20.4 kPa, *p* = 0.01), which reflects that malignant tumors are more heterogeneous in elasticity characteristics. Compared with other methods, the sonographic scoring model has 85% accuracy, and the AUC was 0.82. It combined multiple US characteristics and could be used as a general reference. US-FNA cytology has the highest specificity (93%) and accuracy (91%) among these methods (Table 3). Therefore, US-FNA cytology remains the tool of choice in the evaluation of major salivary gland tumors.

The sonographic characteristics of malignant salivary gland tumors include irregular shape, ill-defined margin, heterogeneous echotexture, absence of posterior echogenicity enhancement, presence of calcification, cystic architecture, larger tumor depth from surface, and presence of regional LN enlargement on B-mode US [2,3,4,20,21]. However, no single parameter has adequate diagnostic accuracy. In our previous study, we reviewed the above characteristics with vascular patterns by power Doppler US, developed a sonographic scoring model for the prediction of malignant salivary gland tumors, and reported the AUC (0.90), sensitivity (70%), specificity (94%), and accuracy (90%). We further validated this prediction model in this study, and it reported good specificity [89% (84% to 95%)] and accuracy [85% (79% to 91%)] with an acceptable AUC (0.82). The slightly poor diagnostic performance might be due to the following reasons. First, this is a different case series from the development population and the inclusion criteria were slightly different. For evaluation the SWE, we excluded those who did not receive SWE in this study. Different case series and different inclusion criteria might result in heterogeneity. Seconds, we used the ATL HDI 5000 (Philips, Bothell, WA, USA) before 2015 and Aplio 500 (Canon Medical Systems, Tochigi-ken, Japan) after 2015. Different US machines might result in different image quality and further affecting the diagnosis. We suggest that this sonographic scoring model could be used to assist in the evaluation of major salivary gland tumors.

The SD of SWE showed a significant difference between benign and malignant salivary gland tumors by using the 5-mm ROI. The application of SD of SWE was mentioned in previous studies. Bhatia et al. [8] first reported the concept of the SD of elasticity, which represents spatial heterogeneity. They reported that the SD of elasticity among 60 salivary gland tumors was highest in mucoepidermoid carcinomas (median, 44.2 kPa), followed by pleomorphic adenomas (median, 12.4 kPa), and other remaining tumors (medians, 1.4–10.3 kPa). However, the ROI in their study was defined as the whole lesion. Wierzbicka et al. [9] also reported that the SD of elasticity was higher in 10 malignant tumors than in 33 benign tumors (mean, 104.7 vs. 48.0 kPa). However, instead of the 5-mm circular ROI in our study, their ROI was defined as a 2-mm circular region, and they put four ROIs in the same picture, with two ROIs in the tumor center and two ROIs in the peripheral region. Heřman et al. [10] revealed that the SD of elasticity was significantly different between benign and malignant parotid gland tumors (*p* = 0.0004). They used four ROIs in the same picture, with the largest ROI covering the entire tumor. The other three ROIs used the preset size, with one in the center, one in the stiffest area, and one in the softest area. However, the preset size was not mentioned in their study, and the SD of elasticity was not clearly informed by using which one of four ROIs. The different ROIs that studies used might result in varying values of SD of elasticity. The 5-mm ROI is commonly used in the evaluation of lymph nodes and thyroid nodules [12,22]. Thus, we used the 5-mm ROI for the evaluation of major salivary gland tumors. Another reason that we did not choose the whole lesion as the ROI was that salivary tumors were near the mandible bone, which might result in local heterogeneity of elasticity if we placed an ROI near the bone [8]. A previous study showed that for breast lesions, the AUC of the SD of elasticity for diagnosing malignancy is higher in ROIs with larger diameters (3 mm vs. 1 mm) [23]. Whether or not the use of 5-mm ROI is better than 1-mm, 3-mm, or whole lesion ROI needs more studies. Further study is mandatory not only for the survey of optimal ROI in the evaluation of salivary gland lesions but also for the application of the SD of SWE in diagnosing malignant salivary gland lesions.

The average SWE, on the other hand, showed no significant difference between benign and malignant tumors in our study (median ± IQR, 49.4 ± 40.3 vs. 61.8 ± 32.7 kPa, *p* = 0.31, Table 2). The null difference might be due to limited case numbers in the group of malignant tumors (N = 19). Wierzbicka et al. showed that there was a significant difference in the average elasticity between 33 benign and 10 malignant tumors [9]. However, Bhatia et al. showed that there was obvious overlap of the average elasticity between 55 benign and 5 malignant tumors [8]. Wang et al. also reported no significant difference between 46 benign and 10 malignant tumors [11]. Based on current evidence, the role of the average SWE is still controversial.

On the other hand, we did not routinely record the maximum and minimum SWE in this study. Heřman et al. reported significant differences in both maximum and minimum SWE between 96 benign and 28 malignant tumors. However, Wang et al. reported no significant difference between 46 benign and 10 malignant tumors. Although the role of the maximum and minimum SWE is not established, further study may include these parameters to validate its accuracy.

The RTE also showed poor diagnostic accuracy (70%) in our study (Table 3). There were different scoring systems (2 to 5 points) among the studies. The 5-point Tsukuba elasticity scores are widely used in the evaluation of breast lesions [24,25,26]. A score of 1 represents almost soft (almost green within the lesion), a score of 2 represents mostly soft (green more than blue), a score of 3 represents mostly stiff (blue more than green), a score of 4 represents almost stiff (almost blue), and a score of 5 represents stiffness out of the lesion (blue extends out of the lesion). For differentiating between benign and malignant breast lesions, the cutoff value was between 3 and 4. However, since Bhatia et al. first used the RTE with a 4-point scoring system in the evaluation of salivary gland tumors in 2010 [27], most studies used the 4-point grading system later. Li et al. reported six of nine studies using the four grades to evaluate salivary gland tumors in one meta-analysis in 2016 [7]. After 2016, only two English studies used the RTE in the evaluation of salivary gland tumors, and both studies used the 4-point scoring system [28,29]. Thus, we also used the 4-point scoring system in our study. However, most studies used a cutoff value between 2 and 3 for the diagnosis of malignant salivary gland tumors. On the other hand, the 5-point Tsukuba elasticity scores used a cutoff value between 3 and 4 for diagnosing malignant breast lesions. Thus, we further calculated the 4-point system of RTE with a cutoff value between 3 and 4 for diagnosing malignant salivary gland tumors, and the sensitivity, specificity, and accuracy with 95% CI were 25% (4% to 46%), 91% (85% to 97%), and 81% (74% to 89%), respectively. Although better accuracy and specificity were noted compared with cutoff values between 2 and 3, the sensitivity was sacrificed. The poor diagnostic accuracy of RTE with a cutoff value between 2 and 3 might be due to the similar stiffness between malignant tumors and pleomorphic adenomas (Figure 2) [30]. Additionally, the RTE is operator dependent, and different compression pressures may affect the elasticity [31].

For diagnosing malignant salivary gland tumors, US-FNA cytology had the highest specificity (93%) and accuracy (91%) among the methods in this study. However, it is an invasive procedure compared with the other three methods and may result in complications [32]. The most serious complications include tumor seeding through the needle track and transient facial paralysis. Shah et al. [33] reported that the tumor seeding rate of FNA was approximately 0.01% in a review study. Although the incidence rate is rare, we still need to be aware of this possibility and know how to manage this complication. On the other hand, transient facial paralysis is an extremely rare complication after FNA. Only one case was reported in the literature [34]. Another important issue is that the diagnosis of cytology depends on the experience of pathologists and has the inter-institutional variability. Although the risk of malignancy (ROM) of AUS (atypia of undetermined significance) in the Milan System for Reporting Salivary Gland Cytopathology (MSRSGC) was only 20%, several studies showed that their ROM of AUS was higher than 20%, around 47–50% [15,16,17]. Before the proposal of Milan system, the ROM of cytological report with atypia was also around 53%-63% [18,19]. Based on the possibility of high ROM of atypia, we took the atypia as suspicious malignancy on the diagnostic criteria of US-FNA cytology. In our study group, there were nine cases with cytology of atypia. All of these cases received further operations, and the pathology showed 4 of 9 (44%) were malignancy.

Despite the highest specificity and accuracy of US-FNA cytology in our study, the sensitivity was only 74% in the diagnosis of malignant salivary gland tumors. Schmidt et al. [35] reported that the pooled sensitivity and specificity of US-FNA were 80% and 97%, respectively, in a meta-analysis. The possible reason for the low sensitivity includes the diagnostic difficulty of the low-grade malignancy by only FNA cytology [36]. US-guided CNB (US-CNB), on the other hand, has higher sensitivity than US-FNA in the diagnosis of malignant salivary gland tumors. Song et al. showed that the sensitivity for diagnosing salivary gland tumors in CNB and FNAC was 88.2% and 58.2%, respectively (*p* = 0.006) [36]. Kim et al. reported that the pooled sensitivity and specificity of US-CNB were 94% and 98%, respectively, in a meta-analysis [37]. However, due to the larger needle diameter, US-CNB has higher complications than US-FNA, including local hematoma (0.5%) [37], tumor seeding through the needle track (0.1%) [33], and transient facial paralysis (0.05%) [37]. Thus, with proper use of CNB, the diagnostic accuracy for major salivary gland tumors may have benefits without increasing complications. Although that more examinations before surgery may have a more comprehensive understanding of the disease, side effects also accompanied. In our institute, the pre-surgical investigation included US and US-FNA. We usually reserved CNB for those who failed to obtain the diagnosis by FNA or those who could not receive excisional biopsy, which was also suggested by another study [38]. In our study, eight cases only received CNB as the pathological report, including two chronic sialadenitis, two IgG4-associated sialadenitis, three other benign tumors, and one invasive carcinoma.

On the other hand, the pre-surgical evaluation of minor salivary gland by US alone is more difficult due to the location of minor salivary gland. For tumor located within oral cavity, we can use intra-oral US [39]. For tumor outsides oral cavity or for more detail information, MRI or CT is more suitable than US [40].

In our study, there was also a significant difference in the SD of elasticity between PA and WT (median ± IQR, 28 ± 22.4 vs. 13.7 ± 26.4 kPa, *p* < 0.01) (Table 2). Bhatia et al. [8] revealed that for discriminating between PA and WT, the AUC of the standard deviation of elasticity was higher than the average elasticity (0.82 vs. 0.70). However, in our study, the AUC of the SD of elasticity for discriminating between PA and WT was only 0.69. In addition, we could not differentiate either of these two tumors from other BTs (Figure 2). Thus, the clinical use of the SD of elasticity for differentiating PA from WT is still inconclusive.

## 5. Limitations

There were several limitations in this study. First, it was a retrospective study and not a randomized controlled trial. Second, the number of cases was small. Only 138 cases were included, and only 19 cases were malignant. Third, there were heterogeneous methods among the studies. The ROI was set varyingly, either as a 2-mm, 5-mm circular region, or whole lesion. Comparisons with other studies might not be suitable. Fourth, the ROI was applied by visual inspection, and measurement error might exist. Further large-scale studies are mandatory not only for the survey of the optimal ROI but also for the application of the SD of elasticity.

## 6. Conclusions

In this study, we validated the sonographic scoring model in the diagnosis of malignant salivary gland tumors, and it reported good specificity [89% (84% to 95%)] and accuracy [85% (79% to 91%)] with an acceptable AUC (0.82). We suggest that this sonographic scoring model could be used to assist in the evaluation of major salivary gland tumors. For SWE, malignant salivary gland tumors had a higher SD of elasticity than benign tumors, but the accuracy for diagnosing malignancy was not satisfactory. Currently, the elastography alone is not sufficient in differencing between malignant and benign salivary gland tumors. Further study may survey the optimal ROI and the corresponding SD of SWE in the evaluation of salivary gland lesions. US-FNA cytology remains the tool of choice for diagnosing malignant salivary gland tumors.

## Figures and Tables

**Table 1:** Clinical characteristics of the included cases (N = 138).

Demographic Data	Mean (SD) or N (%)
Age, yr	53 (14)
Sex	
Female	55 (40%)
Male	83 (60%)
Side	
Right	77 (56%)
Left	61 (44%)
Location	
Parotid gland	111 (80%)
Submandibular gland	27 (20%)
Tumor size, long axis, cm	2.5 (1.0)
Tumor size, short axis, cm	1.7 (0.7)
Pathological reports	
All benign tumors	119 (86%)
WT	49
PA	48
Other BTs (basal cell adenoma, oncocytoma, hemangioma, chronic sialadenitis, IgG4-associated sialadenitis, etc.)	22
All malignant tumors	19 (14%)
Poorly differentiated/undifferentiated/invasive carcinoma	6
Mucoepidermoid carcinoma	4
Adenoid cystic carcinoma	3
Lymphoepithelial carcinoma	2
Acinic cell carcinoma	1
Carcinoma ex pleomorphic adenoma	1
Metastatic carcinoma	1
Lymphoma	1

Abbreviations: SD, standard deviation; PA, pleomorphic adenoma; WT, Warthin’s tumor; BT, benign tumor.

**Table 2:** Comparison of the average and standard deviation of elasticity.

	by Benign or Mal, Median (IQR)			by PA or WT, Median (IQR)		
	Benign	Malignant	*p* Value	PA	WT	*p* Value
Average elasticity, kPa	49.4 (40.3)	61.8 (32.7)	0.31	60.45 (28.75)	30.9 (43.1)	<0.01 *
Standard deviation of elasticity, kPa	25.9 (25)	34.8 (20.4)	0.01*	28 (22.4)	13.7 (26.4)	<0.01 *

Abbreviations: IQR, interquartile range; mal, malignant; PA, pleomorphic adenoma; WT, Warthin’s tumor. * Statistical significance, *p* < 0.05.

**Table 3:** Comparison of the different methods for diagnosing malignant salivary gland tumors.

Methods	Sensitivity (95% CI)	Specificity (95% CI)	Accuracy (95% CI)
Sonographic scoring model ^†^	58% (34% to 80%)	89% (84% to 95%)	85% (79% to 91%)
Standard deviation of SWE with cutoff value as 31.5 kPa	74% (54% to 94%)	62% (54% to 71%)	64% (56% to 72%)
4-point system of RTE with cutoff value between 2 and 3	69% (46% to 92%)	70% (61% to 79%)	70% (62% to 78%)
US-FNA cytology	74% (54% to 94%)	93% (89% to 98%)	91% (86% to 96%)

Abbreviation: SWE, shear wave elastography; RTE, real-time elastography; US-FNA, ultrasound-guided fine needle aspiration; CI, confidence interval. ^†^ The model is 2.08 × (vague boundary) + 1.75 × (regional lymphadenopathy) + 1.18 × (irregular or speculated shape) + 1.45 × (absence of posterior acoustic enhancement) + 2.4 × (calcification). The cutoff value for classifying malignancy was greater than or equal to 3.

## Data Availability

The dataset is available in the Supplementary Materials.

## Supplementary Materials

The following supporting information can be downloaded at: https://www.mdpi.com/article/10.3390/diagnostics12102488/s1, Table S1: The dataset.
